# From “teaching by word and deed” to “intelligent mentorship”: ethical reconsiderations of AI-enabled medical education — lessons from China

**DOI:** 10.3389/fmed.2025.1754139

**Published:** 2026-01-12

**Authors:** Zhitao Hou, Jing Chen, Hongwei Guo

**Affiliations:** College of Basic Medical and Sciences, Heilongjiang University of Chinese Medicine, Harbin, Heilongjiang, China

**Keywords:** artificial intelligence, educational reform, ethical dilemmas, humanism, medical education

## Abstract

Artificial intelligence (AI), as a major driving force of the Fourth Industrial Revolution, is profoundly reshaping the landscape of medical education. Driven by the extensive use of intelligent algorithms, big data analysis, and virtual simulation, a new “fourth-generation medical education” is taking shape, emphasizing health orientation, interdisciplinary integration, and intelligent empowerment. The application of AI in medical education significantly enhances instructional efficiency and personalization, advancing reforms in lesson planning, curriculum design, and virtual clinical simulation. However, an inherent tension exists between the humanistic nature of medical education and the mechanical logic of AI: its integration into teaching may cause alienation in teacher–student relationships, weakening of medical humanism, and ethical dilemmas such as algorithmic bias and privacy infringement. Taking China’s medical education practices as an example, this paper systematically examines the ethical challenges of AI-enabled medical education and proposes a three-dimensional ethical reconstruction framework: (1) reshaping teacher–student relationships to preserve the balance between teaching and learning; (2) reinforcing medical humanism to safeguard the compassionate essence of education; and (3) improving ethical governance through coordinated efforts among government, society, hospitals, and universities. The sustainable development of AI-empowered medical education lies in upholding the moral essence of “humanity within intelligence,” preserving the warmth of “teaching by word and deed” while integrating technological rationality with humanistic care.

## 1 Introduction

Artificial intelligence (AI) is rapidly redefining global education systems, emerging as a central force in the Fourth Industrial Revolution and the transformation of higher education. The United Nations Educational, Scientific and Cultural Organization (UNESCO, 2021), in its report AI and Education: Guidance for Policy-makers, pointed out that the deep integration of AI is “reshaping teaching models and educational governance systems,” and emphasized that countries should use AI responsibly to promote educational equity and high-quality development. The World Health Organization (WHO, 2023), in Ethics and Governance of Artificial Intelligence for Health, highlights that AI is not only transforming healthcare delivery systems but also reshaping the ethical foundations of medical education, placing new demands on medical training (1). Meanwhile, the Association of American Medical Colleges (AAMC, 2022) emphasizes in its AI Competencies for Medical Education Framework that medical students must develop systematic AI literacy, data ethics awareness, and human–AI collaboration skills to adapt to future intelligent healthcare environments. Consequently, medical education is transitioning from experience-based instruction to smart, data-driven, and integrative pedagogical paradigms. AI technologies, with their strengths in data analytics, virtual simulation, and personalized learning, have injected new momentum into educational reform. However, such technological empowerment does not come without cost. A potential tension exists between AI’s algorithmic logic and the ethical values of medical education. The tension is especially pronounced in medical education, where medicine is not only a scientific discipline but also a humanistic practice; its educational mission is not only to transmit skills but to cultivate compassion. Currently, the key ethical aspects under consideration in AI-enabled medical education include: (1) the transformation and potential dehumanization of teacher–student relationships, (2) the erosion of medical humanism in curriculum and practice, (3) algorithmic bias and its implications for fairness and equity, (4) data privacy and security vulnerabilities affecting students and patients, and (5) the insufficiency of governance and regulatory frameworks to manage these risks effectively. These interrelated dimensions constitute the foundation for ethical reflection in intelligent educational ecosystems.

However, the rapid integration of AI introduces new ethical dilemmas for educators: as algorithms increasingly substitute instructional roles, the traditional model of “teaching by word and example” is being redefined (2, 3). This paper aims to address these concerns by proposing a structured ethical reconstruction along five dimensions: pedagogical relationships, medical humanism, algorithmic fairness, data governance, and ethics governance—together forming a three-dimensional ethical framework for AI-enabled medical education. Using China’s transformation of medical education as a case, this paper explores how to reconstruct ethical order within intelligent teaching systems and achieve the organic unity of technological empowerment and educational essence.

### 1.1 The emergence of AI-driven medical education and the ethical tensions it entails

Artificial intelligence (AI) has been widely applied in global healthcare for individualized treatment planning, drug discovery, disease prediction modeling, and virtual medical services (4, 5). In China, AI has been deployed across clinical inquiry and documentation, drug research and biobank data management, disease screening and precision medicine, clinical trials, and hospital administration, becoming a key driver of healthcare digitalization and intelligent transformation. However, despite significant advancements in medical AI applications, corresponding reforms in medical education have lagged behind, with existing pedagogical structures and ethical oversight failing to fully align with technological progress (2). The misalignment between educational structures and technological innovation has generated a series of ethical dilemmas in AI-enabled medical education, including alienation of teaching relationships, inequities in learning access, erosion of medical humanism, and risks of algorithmic bias and privacy breaches (6, 7).

Currently, many Chinese medical schools have integrated AI-driven teaching development into educational innovation initiatives, forming reform models characterized by intelligent empowerment, data-driven learning, and human–AI collaboration (8). Peking Union Medical College Hospital employs VR-based immersive endoscopic training to enhance anatomical learning and surgical practice of the skull base region. Fudan University offers a course titled “Medical Artificial Intelligence and Machine Learning,” integrating AI technologies into the teaching of medical imaging, omics data, and electronic health records. West China Medical Center of Sichuan University, following the Action Guidelines for Integrating AI Literacy into Undergraduate Education, has developed an intelligent experimental teaching platform and a multimodal clinical skills training system to promote curriculum innovation guided by clinical excellence. AI-enabled medical education has become a key trend in China and reflects a global shift toward intelligent transformation in medical training. However, AI not only reshapes pedagogical models and knowledge dissemination but also introduces systemic challenges to teacher-student relations, humanistic education principles, and ethical governance frameworks (1). Without robust ethical frameworks, AI-driven teaching innovations may blur professional boundaries, obscure algorithmic governance, and weaken humanistic values—constituting a core ethical challenge for AI-enabled medical education.

### 1.2 The transformation of pedagogical relationships: from “teaching by word and deed” to “algorithmic mediation”

With the deep integration of artificial intelligence (AI) technologies into higher education systems worldwide, traditional teacher–student relationships are undergoing profound transformation. Medical education has long been grounded in the principle of “teaching by word and deed,” where instructors serve not only as transmitters of knowledge but also as mentors in clinical experience and professional ethics (9). However, the rapid integration of AI technologies has transformed the educational process into a dual-agent structure of “teacher+AI,” shifting teaching interactions from interpersonal engagement to algorithmic mediation and altering both pedagogical agency and emotional resonance (10, 11).

Under AI-enhanced pedagogical frameworks, teachers are becoming progressively dependent on intelligent systems for curriculum planning, learner behavior analysis, and assessment. AI algorithms can rapidly identify students’ learning trajectories and knowledge gaps, thereby generating personalized instructional plans (12). Simultaneously, pedagogical autonomy is increasingly being transferred from educators to algorithmic mechanisms. When educators rely excessively on algorithmic analysis rather than clinical experience and teacher–student interaction, they risk falling into a state of “algorithmic dependence,” diminishing their agency in guiding students’ intellectual and emotional development (13). For example, in AI-assisted grading systems, algorithms score exams based on standardized answers, overlooking students’ logical reasoning and clinical thinking processes in open-ended responses. Teachers see only algorithm-generated scores, making it difficult to grasp students’ true cognitive processes. Although this data-mediated assessment improves efficiency, it weakens the depth of teacher–student interaction grounded in understanding and feedback (14). As time passes, interpersonal emotional ties in education are diminished; “algorithmic precision” displaces “human empathy,” and the classic mentorship model devolves into a data-driven relationship, eroding the humanistic warmth of medical education. Notably, large language models (LLMs) have already reshaped the structure of pedagogical authority to some extent. Studies have shown that early versions of ChatGPT achieved passing scores on the United States Medical Licensing Examination (USMLE), while its advanced versions even outperformed the average performance of medical students, residents, and practicing physicians in several clinical and professional skill assessments (15). This phenomenon highlights AI’s immense potential in knowledge transmission and skill assessment, but it also raises an educational agency crisis: when AI can explain medical knowledge, answer questions, and provide feedback more precisely, students may begin to question the necessity of human instructors, reducing their reliance on human mentorship (16).

However, it is crucial to recognize that AI, despite its precision and efficiency, lacks the capacity for emotional resonance, ethical intuition, and moral exemplarity that human instructors provide. Medical education is not merely about transmitting clinical facts but about modeling care, compassion, and responsibility—dimensions that require human presence and relational depth. Teaching is a profoundly human act grounded in mentorship, trust, and empathy, which cannot be replaced by algorithms (17, 18). Therefore, educators must proactively reaffirm the irreplaceable role of human mentorship in cultivating the professional identity and moral compass of future physicians.

When teachers rely excessively on AI and overlook emotional engagement, the teacher–student dynamic risks devolving into an “algorithm-mediated interaction,” eroding the trust and warmth essential to education. The primary danger of AI in education stems not from the technology but from the “dehumanization of teaching relationships.” In the humanistic domain of medical education, this relational alienation is particularly sensitive: teaching devoid of emotional resonance and clinical experience cannot truly nurture physicians who embody both technical competence and compassionate care (17, 18). Therefore, the alienation of teaching relationships under AI empowerment essentially reflects the restructuring of educational agency and the erosion of humanistic values. Finding a balance between “intelligent assistance” and “human-centered education” constitutes a central challenge that medical education ethics must urgently address.

### 1.3 The loss of medical humanism: a departure from the core of medical education

The fundamental goal of medical education is not merely the transmission of knowledge and skills, but the cultivation of physicians who possess both compassion and wisdom. Medical humanism, rooted in reverence for life and benevolent care for patients, has evolved through centuries of education and practice to form the ethical foundation of the medical profession (19). Its core principle is “patient-centeredness,” which manifests through respect, empathy, and responsibility in both healing and communication. As emphasized in the Medical Humanistic Care Enhancement Action Plan (2024–2027), medical education should strengthen the cultivation of humanistic spirit throughout teaching, internships, and clinical training, enabling students to grasp the “warmth of medicine” while healing patients.

However, the rapid integration of artificial intelligence (AI) is redefining both the content and methods of medical education. Although AI algorithms can simulate human reasoning and optimize clinical decision-making, their logic remains grounded in probabilistic computation and pattern recognition rather than human emotion, morality, or ethical judgment (20). In other words, AI possesses “cognitive intelligence” but lacks “emotional intelligence.” In the context of medical education, this means that AI cannot convey the empathy and compassion inherent in physician–patient relationships, nor can it replace teachers in nurturing students’ professional ethics and humanistic awareness (21). When medical teaching relies excessively on algorithm-driven knowledge transmission, the educational process gradually loses its humanistic warmth. Students receive more training in data-driven diagnostic models than in ethical reflection and interpersonal empathy (22). AI-generated feedback, while highly accurate, is emotionally inert; virtual simulations can reconstruct clinical contexts but cannot convey how to address a patient’s fear, suffering, or hope. Gradually, medical education risks devolving into a form of “technical rationalism,” producing doctors who are “technically proficient yet emotionally absent”—a trend scholars have labeled the “cold knowledge crisis” of medical training (23). A deeper risk lies in AI’s potential to erode medical students’ ethical sensitivity. Medical humanism demands that physicians balance science with morality and efficiency with compassion in complex contexts, while algorithmic thinking tends to seek the optimal solution rather than the most ethical one. As cautioned by The Lancet Commission on AI in Health (2021): “When medical education is dominated by algorithmic logic, the humanistic judgment and ethical reflection of physicians face systemic decline.” Thus, the greatest challenge of AI-empowered medical education lies not in making technology smarter, but in preventing the “dehumanization” of the educational process (24). Future medical education should rebuild balance between the efficiency brought by AI and the humanistic concern central to medicine, through curriculum design, ethics education, and teacher training, to ensure that the core value of medicine—“human-centeredness”—is not replaced by algorithmic logic. Only in this way can medical education safeguard its humanistic soul in an era of technologically enhanced medicine.

### 1.4 Erosion of individual agency: ethical challenges posed by algorithmic bias and data vulnerabilities

Artificial intelligence-enabled medical education was initially envisioned to enhance teaching innovation by enabling a transition from traditional pedagogy to data-driven precision learning (25). However, when educational processes become deeply dependent on algorithmic logic, their inherent opacity and potential injustice gradually emerge. AI systems make decisions based on historical datasets and algorithmic modeling that frequently encode societal biases and structural inequities (26, 27). Within medical education, these biases can result in unequal resource distribution, distorted assessment outcomes, and constraints on instructional autonomy, thereby producing ethical risks and infringement of individual rights.

First, algorithmic bias fundamentally undermines fairness in medical education. Studies indicate that AI systems frequently absorb implicit biases related to gender, race, and geography at the training stage. When such flawed algorithms are incorporated into teaching, medical students may unconsciously adopt and reproduce distorted clinical perceptions, leading to differential treatment in future practice. Second, the emergence of information cocoons causes the cognitive boundaries of teachers and students to be reshaped by algorithms. AI systems optimize content recommendations based on user preferences, yet this may lead to closed-loop knowledge environments (28). Consequently, educators and learners are restricted by algorithmically curated content, limiting their autonomy in knowledge exploration and critical reasoning. Third, risks to data privacy and information security are becoming increasingly prominent. AI-enabled medical education typically requires the collection of large amounts of personal data, including students’ learning trajectories, assessment records, experimental outcomes, and patient clinical information. Without adequate data governance and encryption safeguards, such information is highly vulnerable to leakage and misuse. Studies have shown that inadequate regulation of educational AI systems can lead to the leakage of sensitive information via cloud platforms, training pipelines, or third-party integration channels (29). Particularly in medical education, if clinical teaching platforms are compromised, not only will student and faculty data be exposed, but patient privacy may also be jeopardized (30). This not only violates the medical ethical principles of “respect for autonomy” and “confidentiality,” but also challenges the “data subject rights” emphasized by international regulations such as the General Data Protection Regulation (GDPR) (31, 32).

**FIGURE 1:**
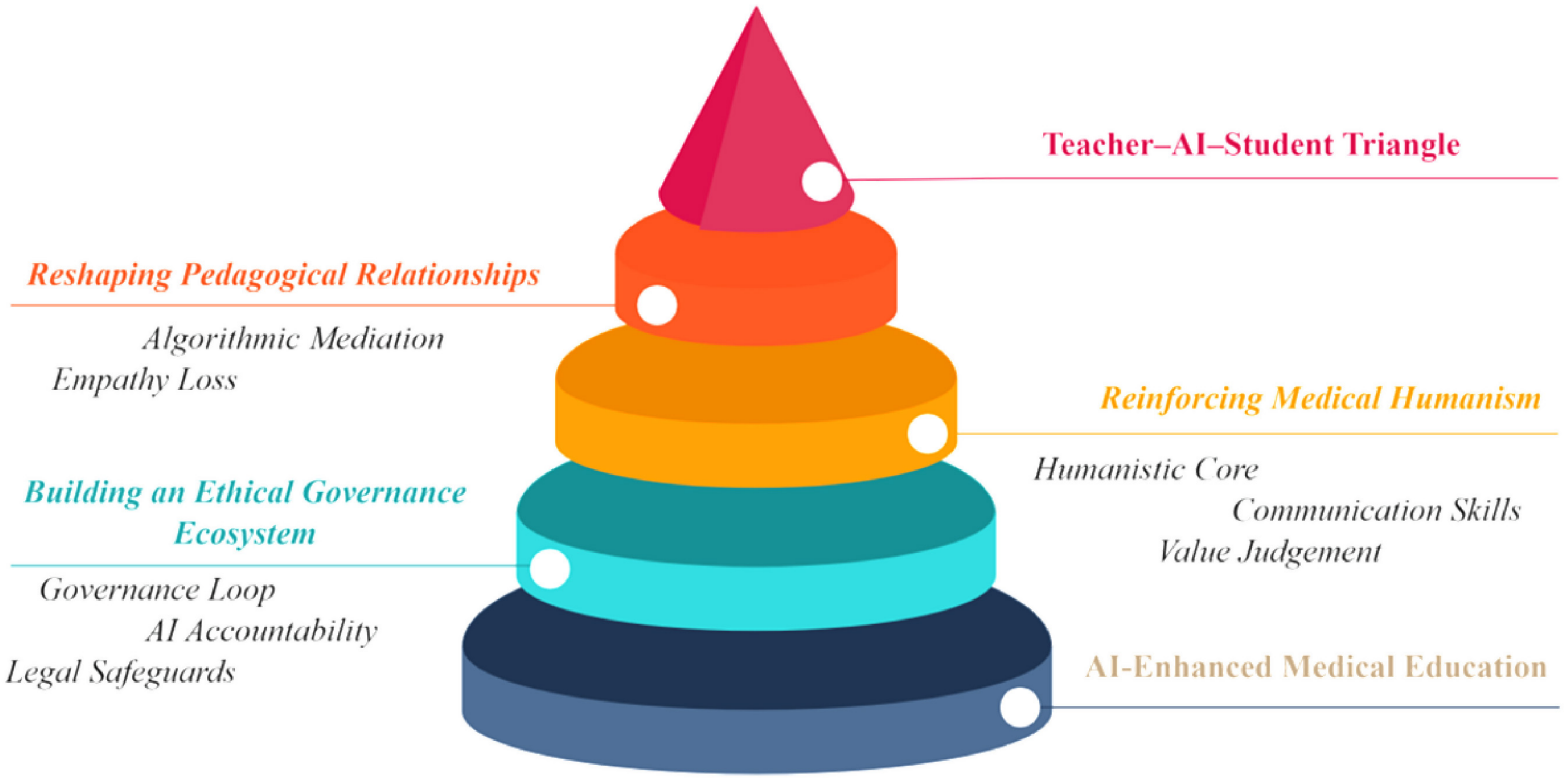
A three-dimensional ethical framework for artificial intelligence (AI)-enhanced medical education: pedagogical relationships, medical humanism, and governance ecosystems.

The ethical challenges posed by AI-enabled medical education concern not only technological controllability but also the rights and dignity of educational participants. When algorithmic logic overrides human judgment, teachers and students can shift from active participants to passive subjects shaped by algorithmic output, thereby destabilizing the ethical foundations of medical education (33). Future medical education should strengthen data governance, algorithmic transparency, and human-centered oversight at the policy level to ensure fairness, trustworthiness, and accountability in intelligent education. Only then can we ensure an educational landscape in which “AI serves humans, rather than humans serving AI” (Figure 1).

## 2 Strategies for addressing ethical dilemmas in AI-enabled higher medical education

### 2.1 Reconfiguring teacher–student identities to reinforce educational equilibrium

Artificial intelligence (AI) technologies have profoundly influenced teacher–student relationships in traditional higher medical education. With the integration of AI, power relations in teaching and patterns of knowledge dissemination have shifted, leading to increasingly indistinct role boundaries between educators and learners (34, 35). To rebuild ethical balance in an intelligent educational environment, it is necessary to reconstruct the role identities of teachers and students from a human-centered perspective. Key measures involve improving educators’ AI instructional competence, developing students’ digital learning capacities, and sustaining humanistic care and moral responsibility within teacher–student interactions. Such efforts facilitate the smooth progression of medical education while reducing the likelihood of technological overdependence and ethical dislocation (36).

#### 2.1.1 Establishing specialized training programs for AI-enabled medical education

The true value of medical education lies in cultivating physicians who can understand patients and communicate humanely, rather than merely producing technically skilled operators. AI should be regarded as an assistive tool to enhance efficiency, rather than a substitute for the judgment of physicians and educators (20). In medical education, AI employs Large Language Models (LLMs) to automatically construct biomedical Knowledge Graphs (KGs) from Electronic Medical Records (EMRs), allowing teachers and physicians to save time on information processing and devote more attention to clinical supervision and patient communication (37, 38). This reflects the ethical core of AI-enabled medical education—“using technology to enhance humanity,” rather than “replacing human empathy with algorithms.” To achieve this goal, higher medical education institutions should systematically establish specialized training systems for AI-empowered medical education. Continuous pedagogical training should enable teachers to master the application of AI technologies in instructional design, classroom interaction, course management, and assessment feedback, while strengthening their clinical practice and humanistic communication skills. As AI lacks the capacity to emulate teachers’ experiential judgment and empathy in complex clinical situations, training must focus on enabling educators to sustain agency and ethical guidance within AI-supported teaching contexts (39). Moreover, teacher training should adopt diverse pedagogical models—such as scenario-based teaching competitions, clinical internships, and interdisciplinary seminars—to promote the integration of “technical literacy, humanistic competence, and ethical awareness.” Educational institutions must recognize that the risks of AI in education stem not only from technical errors but also from excessive teacher reliance on algorithms. Therefore, regular specialized training and ethical workshops should be organized to strengthen teachers’ understanding of AI’s boundaries and ethical use. For example, in applying ChatGPT or similar generative AI tools, educators and clinicians must critically evaluate outputs through cross-verification, treating them as secondary aids rather than substitutes for human expertise or experiential reasoning (40).

To cultivate a balanced educational ecosystem, institutions may host expert-led seminars and cross-disciplinary symposia, connecting teachers with AI developers, medical ethicists, and education researchers. Such exchanges help educators grasp both the potential and constraints of AI tools, while improving their adaptive competence in AI-integrated instruction. The ultimate goal should be to achieve an educational paradigm where “AI assists while teachers lead,” ensuring that students continue to experience the humanistic warmth of “teaching by word and deed” even in intelligent environments.

#### 2.1.2 Strengthening digital literacy among medical students

In an era defined by intelligence and data-driven practices, medical education must regard digital literacy as one of the core competencies for medical students (41, 42). The widespread adoption of AI imposes elevated expectations on how students learn, access knowledge, and make ethical decisions. Medical educators should actively guide students in understanding the logic and application boundaries of AI algorithms and help them develop a three-tier structure of digital literacy encompassing technical operation, information discernment, and innovative application. First, AI technical competency refers to the practical ability of medical students to use intelligent systems for data analysis and learning management. Universities may offer an “Introduction to Medical AI” course to provide new students with several weeks of foundational AI instruction, covering algorithm principles, data security, and applied ethics. Through AI platforms, students can organize, retrieve, and categorize large volumes of medical information more efficiently, improving learning efficiency and fostering autonomous learning (43). Meanwhile, integrating collaborative learning with PBL supports the development of clinical reasoning and diagnostic decision-making skills. Second, AI discernment involves students’ critical thinking and judgment when evaluating algorithm-generated information. The “black box” nature of AI means that some outputs are not fully traceable and may contain false, biased, or unverified information. Students must develop the capacity to scrutinize AI outputs, maintaining skepticism toward automated conclusions while adhering to ethical and scientific standards (22). Case-based instruction, ethical dialogues, and scenario-based decision training may be used to cultivate critical data awareness and professional accountability among students. Third, AI innovation capability reflects the ability of medical students to apply AI technologies in medical innovation. AI-driven virtual simulation systems and immersive instructional environments (Virtual and Augmented Reality) provide students with opportunities to practice clinical operations (44). Using such platforms, students gain procedural familiarity and simultaneously learn to evaluate algorithmic constraints, fostering critical and creative reasoning. Future physicians must possess both clinical competence and technological literacy to meet the multidimensional demands of intelligent healthcare systems (45).

#### 2.1.3 Strengthening two-way teacher–student interaction

The widespread adoption of AI may weaken the emotional connection between teachers and students; therefore, medical education must rebuild mechanisms of interaction on the foundation of intelligent teaching. Teachers should actively guide students to understand the assistive nature of AI and correct the misconception that “technology can substitute for learning” (46). Through classroom communication and emotional engagement, teachers can not only assess students’ knowledge comprehension but also understand their psychological state and learning motivation, thereby maintaining a humanistic orientation in education. In clinical education, teachers should strengthen an interactive mechanism of “experience transmission, value guidance, and emotional resonance.” For example, during surgical mentoring or case discussions, teachers can use AI systems to provide real-time data support while fostering students’ critical clinical thinking through questioning and reflective discussion (39). This “human–AI collaborative” teaching model enhances learning outcomes while preserving the humanistic spirit of traditional medical education. In addition, classroom innovation should emphasize student-centered pedagogy, creating collaborative, case-based learning environments. Teachers can utilize AI-assisted classroom feedback systems and virtual simulation platforms to encourage students to pose questions, analyze data, and participate in discussions in real or simulated contexts, forming a bidirectional learning loop (47). The emerging educational paradigm must strike a balance between teacher authority and student autonomy, ensuring that “AI-assisted instruction” evolves into a true pathway for “AI-enhanced humanistic education” (Table 1).

**TABLE 1:** Ethical challenges and countermeasures in artificial intelligence (AI)-enabled medical education in China.

Thematic category	Subtopic	Manifestations/challenges	Response strategies	References
Pedagogical relationships	Transformation of teaching roles	With AI integration, teachers are no longer the sole source of knowledge. Traditional “teaching by word and deed” is challenged, requiring a shift from authoritative instruction to collaborative facilitation.	1. Teacher Training: Enhance AI literacy to enable teachers to guide AI-augmented instruction.	(1, 2, 4, 9–11, 34, 36, 38, 42, 43)
			2. Role Restructuring: Emphasize teachers’ humanistic care and exemplary roles to build a “teacher–student–machine” triadic model.	
	Erosion of Teacher–Student Interaction	AI provides personalized feedback, which may reduce face-to-face interaction and lead to emotional distancing.	1. Strengthen Face-to-Face Engagement: Prioritize direct interaction in courses and clinical practice.	(3–6, 9, 14, 18, 19, 39, 44, 48)
			2. Co-Learning: Encourage joint AI usage to maintain mutual trust.	
Medical humanities	Decline in doctor–patient communication skills	Reduced emphasis on simulated communication leads to insufficient training in empathy and clinical dialogue.	1. Simulated training: expand clinical communication practice via real or virtual patient scenarios.	(5–8, 13, 45, 49–52)
			2. Humanities curriculum: integrate doctor–patient communication and empathy into compulsory coursework.	
	Academic integrity and trust	Overreliance on generative AI may lead to plagiarism or academic misconduct; opacity in AI decision-making may reduce trust in knowledge systems.	1. D28 ethics education: reinforce integrity and critical thinking in medical curricula.	(7, 8, 16, 17, 22, 36, 38, 40, 42, 53)
			2. Technical oversight: use originality check tools and AI-use protocols to foster transparency and collaboration.	
Algorithmic bias	Dataset bias	AI systems may reflect societal biases (e.g., gender, race), resulting in discriminatory teaching or diagnostic outcomes.	1. Data auditing: ensure training datasets are diverse and balanced.	(9–11, 26, 27, 33, 34, 36, 54)
			2. Explainability: promote interpretable AI to support pedagogical decisions.	
			3. Ethical review: remove problematic algorithmic factors via review boards.	
	Lack of algorithmic transparency	“Black-box” decision-making reduces algorithm interpretability, increasing misuse and overreliance.	1. Algorithm oversight: introduce third-party audits and certification standards.	(11, 12, 26, 30, 33, 35, 54)
			2. Feedback channels: establish user reporting systems to correct AI errors or bias.	
Data security	Privacy and data leakage	Student and patient data within AI systems face risks of unauthorized collection, leakage, or misuse.	1. Privacy protection: apply data anonymization and encryption protocols.	(13, 14, 29–31, 32, 41)
			2. Access control: restrict access rights and application scenarios.	
			3. Safety Training: Educate teachers and students on secure AI usage.	
	Data ownership and ethics	Unclear data ownership with platform providers can lead to commercial or ethical disputes.	1. Policy frameworks: clarify data ownership and usage rights with regulatory oversight.	(15, 16, 24, 29, 31, 32, 54)
			2. Ethical guidelines: issue ethics codes for AI use in medical education.	
Ethical governance	Lagging regulation	Lack of sector-specific AI education guidelines hinders ethical compliance.	1. Standards development: introduce institutional and national-level ethical policies.	(17, 18, 20, 24, 36, 47, 55)
			2. Cross-sector collaboration: encourage cooperation among education, health, and tech sectors.	
			3. Teacher–student participation: involve users in policy-making and ethical awareness.	
	Deficit in ethics education	Medical curricula lack sufficient content in AI ethics and philosophy of technology.	1. Curriculum design: add dedicated AI ethics courses and critical reflection modules.	(19, 20, 22, 36, 47, 53, 56)
			2. Professional conduct: reinforce educator ethics to serve as role models for students.	

### 2.2 Reinforcing humanism: restoring the humanistic core of medical education

As AI technologies are increasingly embedded in medical training, they risk shifting the educational focus away from the core ethical and humanistic values that define the profession—compassion for life, respect for individual dignity, and the moral agency of physicians. These values are especially vulnerable when human instruction is replaced by algorithmic feedback and virtual simulations. To counteract this, medical educators must actively reconstruct an ethical-pedagogical balance, ensuring that technology serves, rather than supplants, the development of humane physicians. This reconstruction entails explicit reinforcement of humanistic competencies such as empathy, patient-centered communication, and professionalism, as well as systematic cultivation of ethical reasoning. The latter involves guiding students to critically evaluate morally complex scenarios introduced by AI, including algorithmic bias, opaque decision-making, data ownership, and fairness in clinical recommendations (48). Ethical reasoning in this context includes four key dimensions: (1) recognizing and responding to the ethical implications of AI use; (2) balancing competing principles such as beneficence, autonomy, and justice; (3) scrutinizing the normative assumptions embedded in algorithms; and (4) making morally justified decisions in ambiguous or resource-limited clinical contexts.

To implement this, pedagogical methods such as ethics-infused problem-based learning (PBL), simulated ethical scenarios, reflective journaling, and interdisciplinary AI ethics seminars are being integrated into medical curricula. These initiatives empower students to interpret AI not merely as a tool but as a moral actor whose influence must be mediated by professional judgment. Ultimately, restoring the ethical and humanistic core of medical education is not a rejection of intelligent systems, but a deliberate act of curricular stewardship—ensuring that technological progress enhances, rather than erodes, the physician’s moral compass.

#### 2.2.1 Strengthening humanistic education: upholding the “patient-centered” principle

The “patient-centered” model is a cornerstone of modern medicine, emphasizing the patient’s quality of life and holistic experience rather than the disease alone (49). Although AI offers powerful data analytics and diagnostic support, the emotional understanding, empathetic communication, and trust-building essential to doctor–patient relationships still depend on healthcare professionals (50). Therefore, medical education must enhance students’ empathy, communication, and emotional support skills through systematic training. In clinical practice, medical students should integrate AI-generated diagnostic suggestions with their medical knowledge while respecting patients’ values, contexts, and autonomy. Compassionate communication can ease patient anxiety, strengthen therapeutic trust, and enhance recovery outcomes and satisfaction. In this process, technology provides information and structural support, whereas humanistic ability determines the warmth and quality of medical practice.

#### 2.2.2 Creating humanistic learning scenarios: adhering to the Principle of Beneficence

The “Principle of Beneficence” is a core tenet of medical ethics, emphasizing that all medical decisions should aim to protect and promote patient welfare (51). In AI-assisted decision-making scenarios, when algorithmic recommendations conflict with clinical realities or patient preferences, medical students must exercise independent judgment. Using AI-enabled platforms such as Virtual Reality (VR), Clinical Scenario Simulation (CSS), and Scenario-Based Ethical Reasoning Training, educators can build immersive ethical learning environments where students experience “decision–evaluation–reflection” cycles safely (52). For example, in ethical situations involving “limited medical resources,” “decisions on invasive resuscitation,” or “treatment conflicts due to financial constraints,” medical students must balance clinical conditions, therapeutic value, quality of life, family wishes, and ethical norms. These ethical norms include the principles of beneficence (doing good), non-maleficence (avoiding harm), justice (fair distribution of resources), and respect for patient autonomy. Together, they form the foundational framework guiding students’ moral reasoning and deontological understanding in clinical decision-making (51). This enhances ethical sensitivity and fosters a sense of independent decision-making responsibility.

#### 2.2.3 Broadening humanistic curriculum: establishing medical ethics as the Core of education

Medical professionalism is the moral foundation of medical education, reflecting physicians’ respect for life, responsibility toward patients, and professional self-discipline (53). AI-driven medical education does not diminish moral education; rather, it demands a more systematic construction of humanistic and ethical curricula for medical students. Instructors can employ AI’s analytical capacity to systematize resources such as medical history, ethical case studies, narrative medicine archives, and communication simulations, creating a dynamic humanistic learning repository. Additionally, strategies such as reflective writing, patient-narrative learning, situational communication training, and peer feedback can be incorporated into curricula to help students internalize professionalism through lived experience. The ultimate goal of medical education is to cultivate “complete physicians” equipped with professional competence, ethical sensitivity, and humanistic compassion. While AI can enhance efficiency and accuracy, the humanistic values of medicine must be continually safeguarded through education itself.

### 2.3 Strengthening the ethical ecosystem: protecting the legitimate rights and interests of all stakeholders

The ethical risks of artificial intelligence (AI) in medical education stem not from the technology itself, but from how it is applied, governed, and value-oriented. Therefore, establishing a sound ethical environment is key to ensuring the healthy development of medical education. Integrating AI into medical education necessitates comprehensive institutional design, algorithmic governance, and multi-stakeholder oversight to safeguard participants’ rights—ensuring technology serves humanity rather than supplants it (54). Furthermore, embedding ethical content into instructional design can enhance students’ ethical sensitivity and moral reasoning. For example, embedding staged ethical decision-making, role-play, and interactive storytelling into AI-driven gamified learning platforms can heighten medical students’ engagement with ethical challenges and facilitate the internalization of moral cognition (56).

#### 2.3.1 Improving the legal and regulatory framework

To prevent AI algorithms from infringing upon the rights of students, educators, and patients, it is essential to strengthen legal and regulatory systems. Globally, AI governance is shifting from technical supervision toward “human-centered, responsible governance.” For example, the EU Artificial Intelligence Act adopts a risk-based framework to regulate the design, deployment, and use of AI, designating healthcare and education as high-risk areas that require algorithmic explainability, transparency, and accountability (European Commission, 2023). Countries such as South Korea and Canada have also emphasized strengthening patient privacy protection, algorithm transparency, and ethical training in the development of medical AI. China has gradually established a regulatory framework for AI governance; the Interim Measures for the Administration of Generative AI Services specify algorithm design, data security, content labeling, and liability boundaries, providing legal foundations for responsible AI use. The Beijing Guidelines for the Application of AI in Education further outline institutional responsibilities, including data usage review, privacy safeguards, and risk response mechanisms. In the future, a unified national framework for AI governance in medical education should be established, providing enforceable standards for data compliance, model bias evaluation, ethical review, and accountability tracking to achieve a “usable, controllable, and accountable” educational environment.

#### 2.3.2 Breaking the “information cocoon” of AI algorithms

AI algorithms tend to generate homogenous knowledge recommendations based on historical data and user preferences, leading to “information cocoons” that limit both students’ and teachers’ clinical perspectives and critical thinking development (28). To avoid this issue: diversify algorithmic information sources—institutions should require AI systems to integrate interdisciplinary, cross-professional, and multilingual databases to prevent bias from single-source data. Teachers should participate in the training and validation of AI recommendation models, reviewing, supplementing, and critically interpreting AI-generated content—especially in courses like laboratory medicine, radiology, and evidence-based diagnosis—to guide students in understanding the complexity of knowledge. Establish feedback and correction mechanisms: when AI outputs incomplete, biased, or outdated information, teachers and students should collaboratively fine-tune the model through interactive feedback. Expanding knowledge structures and maintaining content diversity can not only break algorithmic cognitive closure but also help students develop judgment, reflection, and innovation skills for dealing with uncertainty in future clinical practice.

#### 2.3.3 Building a multi-stakeholder collaborative supervision model

Given AI systems’ rapid iteration, frequent updates, and complex applications, governance cannot be handled by a single entity but requires multi-institutional collaboration (55). Therefore, a multi-tier regulatory mechanism involving government, healthcare institutions, educational bodies, technology companies, and social organizations should be established. Government agencies should establish frameworks for data security, algorithm transparency, and ethical review; healthcare institutions and universities should oversee AI use in educational settings; companies must ensure model explainability, risk disclosure, and traceability; and civil society and the public should engage in ethical dialogue and oversight. For example, UNESCO’s global Guidelines for the Use of Generative AI in Education emphasize that educational institutions must establish sustainable oversight, risk assessment, and ethical accountability systems to ensure AI use aligns with fairness, inclusivity, and human dignity. In this collaborative governance framework, “explainability, transparency, and accountability” should serve as the core principles for AI applications in medical education. Only by ensuring a robust oversight system can we prevent the misuse of technology or the erosion of medical ethics by commercial interests, allowing AI to truly advance medical education rather than become a new source of risk.

## 3 Conclusion

In summary, the rapid development of artificial intelligence (AI) has brought unprecedented opportunities and challenges to medical education. On the one hand, the introduction of AI has driven profound innovations in teaching models, resource utilization, and learning pathways, ushering medical education into a new era of intelligence and precision. On the other hand, the algorithmic logic, human–machine dynamics, and ethical boundaries of AI remain ambiguous, giving rise to emerging ethical risks such as alienated teaching relationships, weakened humanism, and erosion of individual rights. To ensure the healthy development of AI-enabled medical education, a balance must be struck between human values and technological advancement. First, it is essential to respect the autonomy of teachers and students, restore ethical balance in teaching relationships, and enhance AI literacy and digital competence so that human interaction and educational warmth are preserved within intelligent environments. Second, medical humanism should be reinforced—ensuring that core principles such as “patient-centeredness,” the “Principle of Beneficence,” and “medical professionalism” remain the spiritual foundations of AI-era medical education, preventing its reduction to mere instrumentalism. Finally, the ethical governance and oversight mechanisms should be improved to create a fair, transparent, and accountable technological environment, where laws and social co-governance safeguard the rights and dignity of all educational participants.

The ethical value of AI depends on how humanity chooses to use it. The ultimate goal of medical education is not for AI to replace teachers or physicians, but to assist them in becoming wiser and more empathetic educators and healers. Only under the guidance of ethical reflection can AI-enabled medical education truly achieve “intelligence in the service of benevolence,” fostering students’ holistic growth, safeguarding patient welfare, and advancing healthcare toward a more human-centered, intelligent, and sustainable future.
